# Enrichment of Circular Code Motifs in the Genes of the Yeast *Saccharomyces cerevisiae*

**DOI:** 10.3390/life7040052

**Published:** 2017-12-03

**Authors:** Christian J. Michel, Viviane Nguefack Ngoune, Olivier Poch, Raymond Ripp, Julie D. Thompson

**Affiliations:** Complex Systems and Translational Bioinformatics, ICube, University of Strasbourg, CNRS, 300 Boulevard Sébastien Brant, 67400 Illkirch, France; vivianengoune@gmail.com (V.N.N.); olivier.poch@unistra.fr (O.P.); raymond.ripp@unistra.fr (R.R.); thompson@unistra.fr (J.D.T.)

**Keywords:** circular code motifs, yeast *Saccharomyces cerevisiae*, gene enrichment

## Abstract

A set X of 20 trinucleotides has been found to have the highest average occurrence in the reading frame, compared to the two shifted frames, of genes of bacteria, archaea, eukaryotes, plasmids and viruses. This set X has an interesting mathematical property, since X is a maximal $C^{3}$ self-complementary trinucleotide circular code. Furthermore, any motif obtained from this circular code X has the capacity to retrieve, maintain and synchronize the original (reading) frame. Since 1996, the theory of circular codes in genes has mainly been developed by analysing the properties of the 20 trinucleotides of X, using combinatorics and statistical approaches. For the first time, we test this theory by analysing the X motifs, i.e., motifs from the circular code X, in the complete genome of the yeast *Saccharomyces cerevisiae*. Several properties of X motifs are identified by basic statistics (at the frequency level), and evaluated by comparison to R motifs, i.e., random motifs generated from 30 different random codes R. We first show that the frequency of X motifs is significantly greater than that of R motifs in the genome of *S. cerevisiae*. We then verify that no significant difference is observed between the frequencies of X and R motifs in the non-coding regions of *S. cerevisiae*, but that the occurrence number of X motifs is significantly higher than R motifs in the genes (protein-coding regions). This property is true for all cardinalities of X motifs (from 4 to 20) and for all 16 chromosomes. We further investigate the distribution of X motifs in the three frames of *S. cerevisiae* genes and show that they occur more frequently in the reading frame, regardless of their cardinality or their length. Finally, the ratio of X genes, i.e., genes with at least one X motif, to non-X genes, in the set of verified genes is significantly different to that observed in the set of putative or dubious genes with no experimental evidence. These results, taken together, represent the first evidence for a significant enrichment of X motifs in the genes of an extant organism. They raise two hypotheses: the X motifs may be evolutionary relics of the primitive codes used for translation, or they may continue to play a functional role in the complex processes of genome decoding and protein synthesis.

## 1. Introduction

The same set X of trinucleotides was identified in genes (reading frame) of bacteria, archaea, eukaryotes, plasmids and viruses [1,2,3]. It contains the 20 following trinucleotides

(1) X={AAC,AAT,ACC,ATC,ATT,CAG,CTC,CTG,GAA,GAC,GAG,GAT,GCC,GGC,GGT,GTA,GTC,GTT,TAC,TTC}

and codes the 12 following amino acids

(2) {Ala,Asn,Asp,Gln,Glu,Gly,Ile,Leu,Phe,Thr,Tyr,Val}.

This set X has several strong mathematical properties. In particular, it is self-complementary, i.e., 10 trinucleotides of X are complementary to the other 10 trinucleotides of X, e.g., AAC∈X is complementary to GTT∈X, and it is a circular code. A circular code is defined as a set of words such that any motif obtained from this set allows to retrieve, maintain and synchronize the original (construction) frame. Motifs from the circular code X (denoted (1) above) having this frame retrieval property are called X motifs. The circular code X is self-complementary but also maximal, i.e., X cannot be contained in circular codes of larger sizes (with strictly more than 20 trinucleotides), and $C^{3}$ (explained below). During the last 20 years, the combinatorial properties of circular codes have been studied in-depth, especially circular codes on the 4-letter alphabet with uniform words of length 2 (dinucleotides, e.g. [4,5]), 3 (trinucleotides, e.g. [6,7]), or any given length [8].

In this article, we describe for the first time an application of the circular code theory to the complete genome sequence of a living organism, namely the eukaryote *Saccharomyces cerevisiae.* The budding yeast *S. cerevisiae* was chosen because it has been a “model” organism for many years and has largely contributed to our understanding of eukaryotic genome evolution [9]. The *S. cerevisiae* genome is a eukaryotic genome, the first to be fully sequenced in 1996 [10] and has a smaller genome size compared to human or mouse. In addition, most of the protein-coding genes have a simple intron/exon structure which facilitates the study of the preferential frames of the X motifs. Furthermore, most of the genes are very well annotated in terms of gene expression and protein function [11]. By performing several basic frequency statistics, new properties of X motifs are identified in this genome depending on their localization (non-coding regions and coding regions of genes), their cardinality (trinucleotide composition), their length, their occurrence in the three frames of genes, etc. All these results represent the first evidence for a significant enrichment of X motifs in the genes of this organism. They allowed us to introduce the concept of X genes, i.e., genes with a reading frame retrieval property. Finally, two hypotheses are proposed that may explain our observations.

## 2. Method

### 2.1. Definitions

We recall a few definitions without detailed explanation (i.e., without figures and examples) that are necessary for understanding the main properties of the X motifs obtained from the trinucleotide circular code X identified in genes [1,2,3].

**Notation** **1.** *Let us denote the nucleotide 4-letter alphabet* B={A,C,G,T} *where* A *stands for adenine,* C *stands for cytosine,* G *stands for guanine and* T *stands for thymine. The trinucleotide set over* B *is denoted by* $B^{3}=\left\{AAA,\ldots ,TTT\right\}$*. The set of non-empty words (words, respectively) over* B *is denoted by* $B^{+}$ *(*$B^{*}$, respectively).

**Notation** **2.** *Genes have three frames* f*. By convention here, the reading frame* f=0 *is established by a start trinucleotide* ATG*, and the frames* f=1 *and* f=2 *are the reading frame* f=0 *shifted by one and two nucleotides in the* 5′−3′ *direction (to the right), respectively.*

Two biological maps are involved in gene coding.

**Definition** **1.** *According to the complementary property of the DNA double helix, the nucleotide complementarity map* C:B→B *is defined by* C(A)=T, C(C)=G, C(G)=C, C(T)=A. *According to the complementary and antiparallel properties of the DNA double helix, the trinucleotide complementarity map* $C:B^{3}\to B^{3}$ *is defined by* $C\left(l_{0}l_{1}l_{2}\right)=C\left(l_{2}\right)C\left(l_{1}\right)C\left(l_{0}\right)$ *for all* $l_{0},l_{1},l_{2}\in B$. *By extension to a trinucleotide set* S*, the set complementarity map* $C:\mathbb{P}\left(B^{3}\right)\to \mathbb{P}\left(B^{3}\right)$, ℙ *being the set of all subsets of* $B^{3}$*, is defined by* $C\left(S\right)=\left\{v:u,v\in B^{3},u\in S,v=C\left(u\right)\right\}$.

**Example** **1.** C({CGA,GAT})={ATC,TCG}.

**Definition** **2.** *The trinucleotide circular permutation map $P:B^{3}\to B^{3}$ is defined by $P(l_{0}l_{1}l_{2})=l_{1}l_{2}l_{0}$ for all* $l_{0},l_{1},l_{2}\in B$. $P^{2}$ *denotes the 2nd iterate of P.* *By extension to a trinucleotide set* S*, the set circular permutation map $P:\mathbb{P}\left(B^{3}\right)\to \mathbb{P}\left(B^{3}\right)$ is defined by* $P\left(S\right)=\left\{v:u,v\in B^{3},u\in S,v=P\left(u\right)\right\}$.

**Example** **2.** P({CGA,GAT})={ATG,GAC} *and* $P^{2}\left(\left\{CGA,GAT\right\}\right)=\left\{ACG,TGA\right\}$.

**Definition** **3.** *A set* $S\subseteq B^{+}$ *is a code if, for each* $x_{1},\ldots ,x_{n},y_{1},\ldots ,y_{m}\in S$*,* n,m≥1*, the condition* $x_{1}\ldots x_{n}=y_{1}\ldots y_{m}$ *implies* n=m *and* $x_{i}=y_{i}$ *for* i=1,…,n*.*

**Definition** **4.** *Any non-empty subset of the code* $B^{3}$ *is a code and called trinucleotide code* C*.*

**Example** **3*.*** The genetic code is a code from a code theory point of view.

**Definition** **5.** *A trinucleotide code* $C\subseteq B^{3}$ *is self-complementary if, for each* t∈C, C(t)∈C*, i.e.,* C=C(C).

**Example** **4.** *The genetic code is a self-complementary code*.

**Definition** **6.** *A trinucleotide code* $X\subseteq B^{3}$ *is circular if, for each* $x_{1},\ldots ,x_{n},y_{1},\ldots ,y_{m}\in X$, n,m≥1, $r\in B^{*}$, $s\in B^{+}$*, the conditions* $sx_{2}\ldots x_{n}r=y_{1}\ldots y_{m}$ *and* $x_{1}=rs$ *imply* n=m, r=ε *(empty word) and* $x_{i}=y_{i}$ *for* i=1,…,n.

**Example** **5.** The genetic code is (obviously) not circular.

We briefly recall the proof used to determine whether a code is circular or not, with the most recent and powerful approach which relates an oriented (directed) graph to a trinucleotide code.

**Definition** **7.** *[8]. Let* $X\subseteq B^{3}$ *be a trinucleotide code. The directed graph* G(X)=(V(X),E(X)) *associated with* X *has a finite set of vertices (nodes)* V(X) *and a finite set of oriented edges* E(X) *(ordered pairs* [v,w] *where* v,w∈X) *defined as follows*:

$$\{\begin{array}{c} V\left(X\right)=\left\{N_{1},N_{3},N_{1}N_{2},N_{2}N_{3}:N_{1}N_{2}N_{3}\in X\right\}\\ E\left(X\right)=\left\{\left[N_{1},N_{2}N_{3}\right],\left[N_{1}N_{2},N_{3}\right]:N_{1}N_{2}N_{3}\in X\right\} \end{array}.$$

The theorem below gives a relation between a trinucleotide code which is circular and its associated graph.

**Theorem** **1.** *[8]. Let* $X\subseteq B^{3}$ *be a trinucleotide code. The following statements are equivalent:*

(i) *The code* X *is circular.*
(ii) *The graph* G(X) *is acyclic.*

**Definition** **8.** *A trinucleotide circular code* $X\subseteq B^{3}$ *is* $C^{3}$ *self-complementary if* X, $X_{1}=P\left(X\right)$ *and* $X_{2}=P^{2}\left(X\right)$ *are trinucleotide circular codes such that* X=C(X) *(self-complementary),* $C\left(X_{1}\right)=X_{2}$ *and* $C\left(X_{2}\right)=X_{1}$ ($X_{1}$ *and* $X_{2}$ *are complementary*).

*The trinucleotide set* $X=X_{0}$ *(1) coding the reading frame (*f=0*) in genes is a maximal (20 trinucleotides)* $C^{3}$ *self-complementary (*X=C(X)*) trinucleotide circular code [3] where the circular code* $X_{1}=P\left(X\right)$ *coding the frame* f=1 *contains the 20 following trinucleotides*

(3) $$X_{1}=\{AAG,ACA,ACG,ACT,AGC,AGG,ATA,ATG,CCA,CCG,\;GCG,GTG,TAG,TCA,TCC,TCG,TCT,TGC,TTA,TTG\}$$

*and the circular code $X_{2}=P^{2}\left(X\right)$ coding the frame* f=2 *contains the 20 following trinucleotides*

(4) $$X_{2}=\{AGA,AGT,CAA,CAC,CAT,CCT,CGA,CGC,CGG,CGT,\;CTA,CTT,GCA,GCT,GGA,TAA,TAT,TGA,TGG,TGT\}.$$

*The trinucleotide circular codes* $X_{1}$ *and* $X_{2}$ *are related by the permutation map, i.e.,* $X_{2}=PX_{1}$ *and* $X_{1}=P^{2}\left(X_{2}\right)$, *and by the complementary map, i.e.,* $X_{1}=C\left(X_{2}\right)$ *and* $X_{2}=C\left(X_{1}\right)$ *[12].*

### 2.2. Definition of X Motifs and Random Motifs

Let a X motif m(X) be a sequence (word) constructed from the circular code X (1). Similarly, we define a R motif m(R) constructed from one of the random codes R given in Appendix A. In order to obtain a statistically significant distribution, a set of |R|=30 random codes R are generated according to the properties of X, except its circularity property:

(i) R has a cardinality equal to 20 trinucleotides;
(ii) The total number of each nucleotide A, C, G and T in R is equal to 15 (note that 20×3=15×4);
(iii) R has no stop trinucleotides {TAA,TAG,TGA} and no periodic trinucleotides {AAA,CCC,GGG,TTT};
(iv) R is not a circular code. Its associated graph G(R) is cyclic (G(R) being not shown).

Each motif, m(X) or m(R), is characterized by its cardinality c in trinucleotides and its length l in trinucleotides.

**Example** **6.** *For the convenience of the reader, we give an example of a motif* $m\left(X\right)=m_{1}$ *from the circular code* X *(1) in a sequence* s*:* …AAAGGTGCCGAAGCCCTGGAGGAAAAG… *In* s*,* *there is a* X *motif* $m_{1}=GGTGCCGAAGCCCTGGAGGAA$ *of cardinality* c=5 *trinucleotides* {CTG,GAA,GAG,GCC,GGT} *and* *length* l=7 *trinucleotides. Note that this motif* $m_{1}$ *cannot be extended to the left or to the right* *in* s *due to the presence of the periodic trinucleotide AAA (left) and the trinucleotide AAG (right) which both do not belong to* X.

*The fundamental property of a motif* m(X) *is the ability to retrieve, synchronize and maintain the reading frame. Indeed, a window of 13 nucleotides located anywhere in a sequence generated from the circular code* X *(1) is sufficient to retrieve the reading (correct, construction) frame of the sequence.*

**Example** **7.** *With the previous example of the* X *motif* $m_{1}$*, the reading frame of the sequence* s *is:*

…,AAA,GGT,GCC,GAA,GCC,CTG,GAG,GAA,AAG,….

It is important to stress again that this window for retrieving the reading frame in a sequence can be located anywhere in the sequence, i.e., no other frame signal, including start and stop trinucleotides, is required to identify the reading frame.

Since a huge number of X motifs m(X) can be identified in a complete genome, we selected specific classes of X motifs, denoted m(X,c), where c=4,…20 is the cardinal in trinucleotides, with any length l≥c≥4 in trinucleotides. Thus, we analyzed 17 classes of motifs m(X,c): m(X,4),…,m(X,20). The minimal length l=4 trinucleotides was chosen based on the requirement for 13 nucleotides in order to retrieve the reading frame. The motifs m(X,c) with cardinality c<4 trinucleotides are excluded here because they are mostly associated with the “pure” trinucleotide repeats often found in non-coding regions of the genome [13].

**Example** **8.** *The previous example of the* X *motif* $m_{1}$ *belongs to the class* m(X,5)*.*

### 2.3. Statistical Analysis of X Motifs in the Genome of S. cerevisiae

Let N(X,c;K) be the occurrence number of the X motifs m(X,c;K) in a sequence population $K=\left\{\mathbb{C},\mathbb{C}H,{\mathbb{C}}_{g},{\mathbb{C}}_{\bar{g}}\right\}$ where K can be the entire genome *S. cerevisiae* K=ℂ, one of its 16 chromosomes K=ℂH, their genes $K={\mathbb{C}}_{g}$ or their non-coding regions $K={\mathbb{C}}_{\bar{g}}$. Similarly, we define N(R,c;K) as the occurrence number of the R motifs m(R,c;K) in K and $\bar{N}\left(R,c;K\right)=N\left(R,c;K\right)/\left|R\right|$ as the mean occurrence number of R motifs m(R,c;K) of the |R|=30 random codes R in K. An X motif or a R motif is considered to belong to a gene ${\mathbb{C}}_{g}$ if at least one trinucleotide of the motif is located within the gene.

### 2.4. Statistical Analysis of X Motifs in the Three Frames of S. cerevisiae Genes

The X motifs in the three frames of genes ${\mathbb{C}}_{g}$ of *S. cerevisiae* were analyzed according to two properties p: their cardinality c and their length l. Let $N\left(X,p,f;{\mathbb{C}}_{g}\right)$ be the occurrence number of the X motifs $m\left(X,p,f;{\mathbb{C}}_{g}\right)$ in the frame f=0,1,2 of genes ${\mathbb{C}}_{g}$. Note that for p=c, $\sum_{f=0}^{2}N\left(X,c,f;{\mathbb{C}}_{g}\right)=N\left(X,c;{\mathbb{C}}_{g}\right)$, $N\left(X,c;{\mathbb{C}}_{g}\right)$ being defined in Section 2.3. We define the proportion $P\left(X,p,f;{\mathbb{C}}_{g}\right)$ of the X motifs $m\left(X,p,f;{\mathbb{C}}_{g}\right)$ in a frame f=0,1,2 of genes ${\mathbb{C}}_{g}$ as $P\left(X,p,f;{\mathbb{C}}_{g}\right)=N\left(X,p,f;{\mathbb{C}}_{g}\right)/N\left(X,p;{\mathbb{C}}_{g}\right)$. Let $\bar{N}\left(R,p,f;{\mathbb{C}}_{g}\right)=N\left(R,p,f;{\mathbb{C}}_{g}\right)/\left|R\right|$ be the mean occurrence number of the R motifs $m\left(R,p,f;{\mathbb{C}}_{g}\right)$ in a frame f=0,1,2 of genes ${\mathbb{C}}_{g}$. Similarly, we define the mean proportion $\bar{P}\left(R,p,f;{\mathbb{C}}_{g}\right)$ of the R motifs $m\left(R,p,f;{\mathbb{C}}_{g}\right)$ in a frame f=0,1,2 of genes ${\mathbb{C}}_{g}$ as $\bar{P}\left(R,p,f;{\mathbb{C}}_{g}\right)=\bar{N}\left(R,p,f;{\mathbb{C}}_{g}\right)/N\left(R,p;{\mathbb{C}}_{g}\right)$.

### 2.5. Statistical Analysis of S. cerevisiae Genes with X Motifs $m_{X}$

A gene, called an X gene, is considered to have an X motif if at least one trinucleotide of the gene belongs to an X motif. Let $N\left({\mathbb{C}}_{g};X,c\right)$ be the occurrence number of X genes ${\mathbb{C}}_{g}$ of *S. cerevisiae* with X motifs $m\left(X,c;{\mathbb{C}}_{g}\right)$. Similarly, we define $N\left({\mathbb{C}}_{g};R,c\right)$ as the occurrence number of genes ${\mathbb{C}}_{g}$ with R motifs $m\left(R,c;{\mathbb{C}}_{g}\right)$ and $\bar{N}\left({\mathbb{C}}_{g};R,c\right)=N\left({\mathbb{C}}_{g};R,c\right)/\left|R\right|$ as the mean occurrence number of genes ${\mathbb{C}}_{g}$ with R motifs $m\left(R,c;{\mathbb{C}}_{g}\right)$ from the |R|=30 random codes R.

As previously, we define the proportion $P\left({\mathbb{C}}_{g};X,c\right)$ of X genes ${\mathbb{C}}_{g}$ with X motifs $m\left(X,c;{\mathbb{C}}_{g}\right)$ as $P\left({\mathbb{C}}_{g};X,c\right)=N\left({\mathbb{C}}_{g};X,c\right)/N\left({\mathbb{C}}_{g}\right)$ where $N\left({\mathbb{C}}_{g};X,c\right)$ is the number of X genes ${\mathbb{C}}_{g}$ (see above) and $N\left({\mathbb{C}}_{g}\right)$ is the total number of genes ${\mathbb{C}}_{g}$ in ℂ (given in Section 2.7). Similarly, we define the mean proportion $\bar{P}\left({\mathbb{C}}_{g};R,c\right)$ of genes ${\mathbb{C}}_{g}$ with R motifs $m\left(R,c;{\mathbb{C}}_{g}\right)$ as $\bar{P}\left({\mathbb{C}}_{g};R,c\right)=\bar{N}\left({\mathbb{C}}_{g};R,c\right)/N\left({\mathbb{C}}_{g}\right)$ where $\bar{N}\left({\mathbb{C}}_{g};R,c\right)$ is the mean occurrence number of genes ${\mathbb{C}}_{g}$ with R motifs $m\left(R,c;{\mathbb{C}}_{g}\right)$ and $N\left({\mathbb{C}}_{g}\right)$ is the total number of genes ${\mathbb{C}}_{g}$ in ℂ (given in Section 2.7).

### 2.6. Software Development

A program was developed in the Java language to identify X and R motifs in all 3 frames of an input nucleotide sequence [13]. The program takes optional parameters that define the minimum cardinality c (in trinucleotides) and the length l (in trinucleotides) of the X motifs searched, as well as the trinucleotides making up the X or R code. It returns a list of all X or R motifs identified within the sequence, including the motif sequence, length, cardinality and frame.

### 2.7. Genome S. cerevisiae

The reference genome ℂ of *S. cerevisiae* strain S288C (version R64-2-1) and gene annotations were downloaded from Ensembl (http://www.ensembl.org/, June 2017). The genome contains 13,986,094 nucleotides and a total number of $N\left({\mathbb{C}}_{g}\right)=6691$ genes, whose coding regions represent 8,997,548 nucleotides (64.3% of the genome).

**Figure 1:**
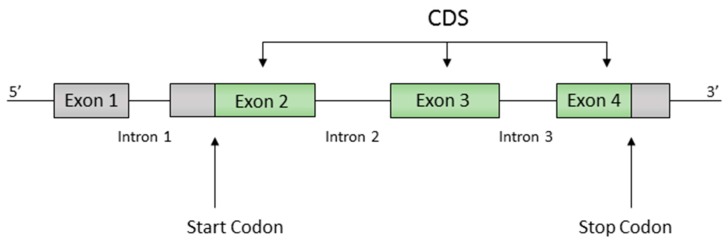
Example of a gene structure, showing exons, introns and the CoDing Sequence (CDS) between the start and stop trinucleotides.

Gene annotations included the positions of all protein coding regions (or CDS for CoDing Sequence), with exons, introns, start codons and stop codons identified. Of the 6691 genes, 6407 genes have a single exon, while 284 genes have a more complex structure with multiple exons separated by one or more introns (Figure 1). In both cases, the CDS is defined as the exon sequence starting with the start trinucleotide ATG and ending with a stop trinucleotide {TAA,TAG,TGA}.

Functional annotations for the 6691 genes were downloaded from the *Saccharomyces* Genome Database (SGD) (https://www.yeastgenome.org/, June 2017).

## 3. Results

The results presented below are based on basic statistics (elementary frequencies) and their biological significance is clear. In order to evaluate the statistical significance of the different results presented below, we chose an approach that involved comparing the results obtained for the X motifs with those obtained for random R motifs generated by 30 different random codes R (see Section 2.2 and Appendix A). This approach avoids the problems associated with defining statistical hypotheses about the nucleotide composition, the length and the random model of the different regions of the genome. The main disadvantage of our approach is the additional computational resources required to obtain the results for 30 different random codes.

### 3.1. Occurrence Number of X Motifs in the Genome of S. cerevisiae

**Figure 2:**
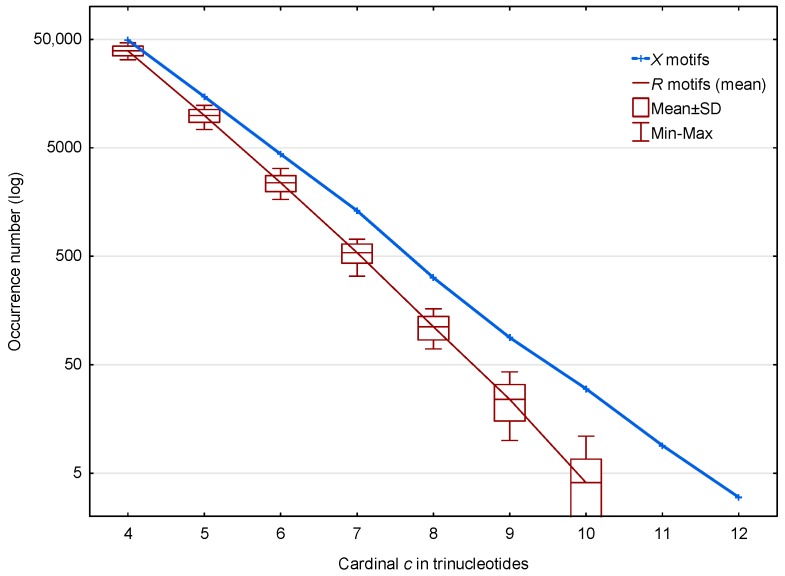
Occurrence number N(X,c;ℂ) (Section 2.3) of X motifs m(X,c;ℂ) (blue) and mean occurrence number $\bar{N}\left(R,c;\mathbb{C}\right)$ (Section 2.3) of R motifs m(R,c;ℂ) (red) in the genome ℂ of *S. cerevisiae*. The abscissa shows the cardinality c=4,…,12 in trinucleotides. The ordinate gives the occurrence numbers N and $\bar{N}$ in logarithm.

In the genome of *S. cerevisiae*, 70,204 X motifs (from the circular code X (1)) and a mean number of 52,183 R motifs (from the 30 random codes R) are observed. The distributions of these X and R motifs according to their cardinality (trinucleotide composition) c are shown in Figure 2. The highest cardinality of the *X* motifs observed is c=12 trinucleotides. Regardless of the cardinality c, Figure 2 shows that the occurrence number of X motifs is very significantly larger than the number of R motifs in *S. cerevisiae*. The distribution of the values obtained for the R motifs is indicated by boxplots representing the mean, the standard deviation and the Minimum–Maximum occurrence numbers. Very similar boxplots were obtained using the median and Q1–Q3 quartiles (statistical results not shown).

Based on this preliminary study, we then wanted to know whether the X motifs are uniformly distributed along the genome or enriched in functional regions, such as the genes.

#### 3.1.1. Occurrence Number of X Motifs in the Non-Coding Regions of *S. cerevisiae*

**Figure 3:**
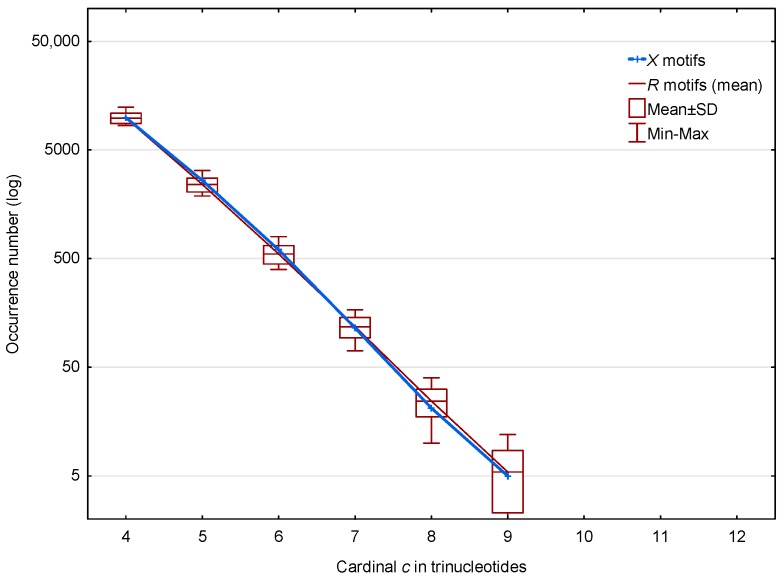
Occurrence number $N\left(X,c;{\mathbb{C}}_{\bar{g}}\right)$ (Section 2.3) of X motifs $m\left(X,c;{\mathbb{C}}_{\bar{g}}\right)$ (blue) and mean occurrence number $\bar{N}\left(R,c;{\mathbb{C}}_{\bar{g}}\right)$ (Section 2.3) of R motifs $m\left(R,c;{\mathbb{C}}_{\bar{g}}\right)$ (red) in the non-coding regions ${\mathbb{C}}_{\bar{g}}$ of *S. cerevisiae*. The abscissa shows the cardinality c=4,…,12 in trinucleotides. The ordinate gives the occurrence numbers N and $\bar{N}$ in logarithm.

In the non-coding regions of *S. cerevisiae*, 13,309 (19.0%) of the X motifs out of 70,204 and 12,936 (mean number) (24.8%) of the R motifs out of 52,183 are observed. The distributions of these X and R motifs according to the trinucleotide cardinality c are given in Figure 3. Regardless of the cardinality c, Figure 3 shows that there is no significance difference between the distributions of the X and R motifs in the non-coding regions of *S. cerevisiae*.

We conclude that the X motifs located in the non-coding regions are random occurrences and are probably not functional. Thus, the differences we observed at the genome level are undoubtedly due to differences in the genes. In the remaining sections of this article, we will concentrate on these important functional regions.

#### 3.1.2. Occurrence Number of X Motifs in the Genes of *S. cerevisiae*

**Figure 4:**
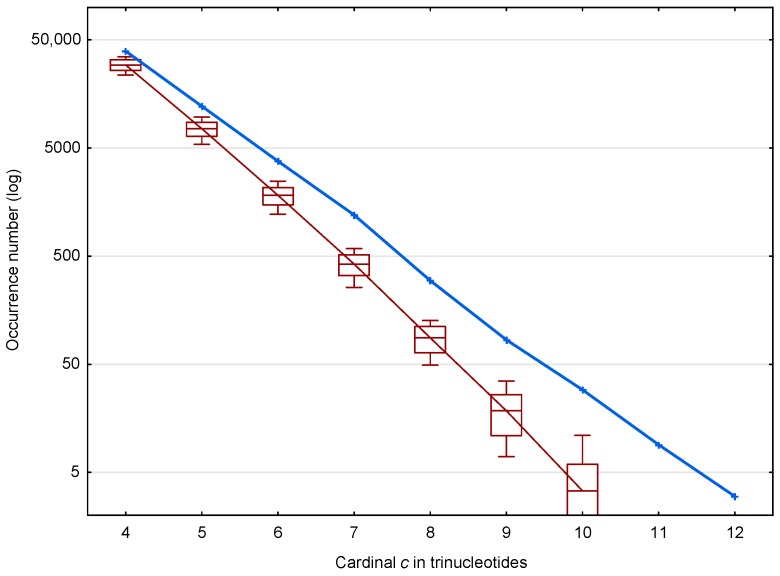
Occurrence number $N\left(X,c;{\mathbb{C}}_{g}\right)$ (Section 2.3) of X motifs $m\left(X,c;{\mathbb{C}}_{g}\right)$ (blue) and mean occurrence number $\bar{N}\left(R,c;{\mathbb{C}}_{g}\right)$ (Section 2.3) of R motifs $m\left(R,c;{\mathbb{C}}_{g}\right)$ (red) in the genes ${\mathbb{C}}_{g}$ of *S. cerevisiae*. The abscissa shows the cardinality c=4,…,12 in trinucleotides. The ordinate gives the occurrence numbers N and $\bar{N}$ in logarithm.

In the coding regions of the genes of *S. cerevisiae*, 56,895 (81.0%) of the X motifs out of 70,204 and 39,247 (mean number) (75.2%) of the R motifs out of 52,183 are identified. The distribution of these X and R motifs according to the trinucleotide cardinality c are given in Figure 4. As expected, important differences are observed in the occurrence numbers of X and R motifs and this is true for all cardinalities from 4 to 12.

**Figure 5:**
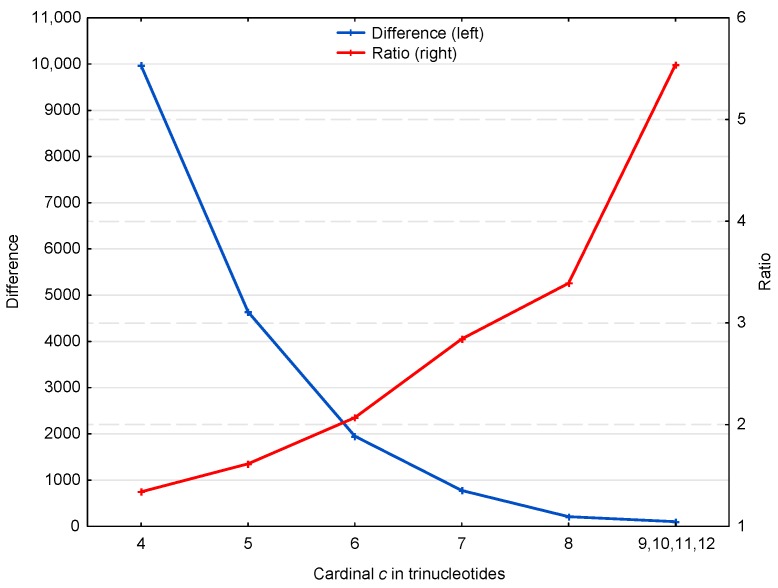
Difference $\delta \left(X,c;{\mathbb{C}}_{g}\right)=N\left(X,c;{\mathbb{C}}_{g}\right)-\bar{N}\left(R,c;{\mathbb{C}}_{g}\right)$ (blue, left) and ratio $r\left(X,c;{\mathbb{C}}_{g}\right)=N\left(X,c;{\mathbb{C}}_{g}\right)/\bar{N}\left(R,c;{\mathbb{C}}_{g}\right)$ (red, right) of X motifs $m\left(X,c;{\mathbb{C}}_{g}\right)$ and R motifs $m\left(R,c;{\mathbb{C}}_{g}\right)$ in the genes ${\mathbb{C}}_{g}$ of *S. cerevisiae* (deduced from Figure 4). The abscissa shows the cardinality c=4,…,12 in trinucleotides. The ordinate gives the occurrence numbers δ and r.

Figure 4 suggests two properties of the X motifs affecting the retrieval of the reading frame in genes, which are represented in more detail in Figure 5. First, the ratio of X motifs to R motifs, i.e., $r\left(X,c;{\mathbb{C}}_{g}\right)=N\left(X,c;{\mathbb{C}}_{g}\right)/\bar{N}\left(R,c;{\mathbb{C}}_{g}\right)$, increases with the trinucleotide cardinality (red curve in Figure 5). At first sight, this might suggest that the X motifs with large cardinalities are more important for retrieving the reading frame in genes. However, it should be noted that these X motifs are relatively rare (131 X motifs of cardinality c=9,10,11,12 trinucleotides) compared to the low cardinality X motifs (49,265 X motifs of cardinality c=4 trinucleotides) (blue curve in Figure 5). Indeed, the second property shows that low cardinality X motifs are highly abundant with ~10,000 more X motifs of cardinality c=4 trinucleotides, for example, than expected by chance. It is important to remember that an X motif of cardinality c=4 trinucleotides, i.e., of length l≥4 trinucleotides, is sufficient to retrieve the reading frame (by definition of a circular code).

**Figure 6:**
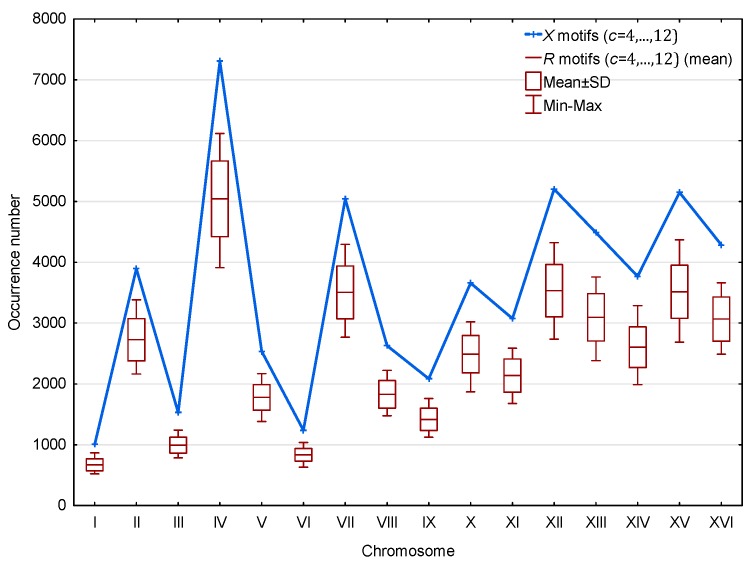
Occurrence number $N\left(X,c\geq 4;\mathbb{C}H_{g}\right)$ (Section 2.3) of X motifs $m\left(X,c\geq 4;\mathbb{C}H_{g}\right)$ (blue) and mean occurrence number $\bar{N}\left(R,c\geq 4;\mathbb{C}H_{g}\right)$ (Section 2.3) of R motifs $m\left(R,c\geq 4;\mathbb{C}H_{g}\right)$ (red) in the genes $\mathbb{C}H_{g}$ of the 16 chromosomes ℂH of *S. cerevisiae*. The abscissa shows the 16 chromosomes. The ordinate gives the occurrence numbers N and $\bar{N}$ in logarithm.

Furthermore, as shown in Figure 6, a significantly large number of X motifs relative to R motifs is observed in the genes $\mathbb{C}H_{g}$ of the 16 chromosomes ℂH of *S. cerevisiae*. This result is statistically significant. Indeed, the probability that a point in the curve of Figure 6 associated with the X motifs is higher than the point associated with the R motifs is equal to 1/2. Then, the probability that the X motifs are more numerous than the R motifs in each of the 16 independent chromosomes is equal to $1/2^{16}\approx 10^{-5}$. Finally, this result is independent of the length or coding gene density of the chromosomes.

Table 1 lists the longest X motifs in the genes of *S. cerevisiae* of length greater than 100 nucleotides. Surprisingly, these X motifs exhibit two fundamentally different structures. The first class consists of X motifs containing a sequence of a repeated trinucleotide ${\left(N_{1}N_{2}N_{3}\right)}^{n}$, e.g., $m_{6}$ with a trinucleotide repeated 20 times, precisely ${\left(ATC\right)}^{20}$. The second class includes X motifs with no repeated trinucleotide (n=1), e.g., $m_{8}$ with 34 trinucleotides not repeated. An intermediary class is composed of X motifs between these two extremes, e.g., $m_{1}$ is composed of a series of different short trinucleotide repeats.

In the next section, we describe a more in-depth statistical analysis of X motifs in genes relative to their frames: the reading frame 0 and its two shifted frames 1 and 2.

### 3.2. Occurrence Number of X Motifs in the Three Frames of S. cerevisiae Genes

**Figure 7:**
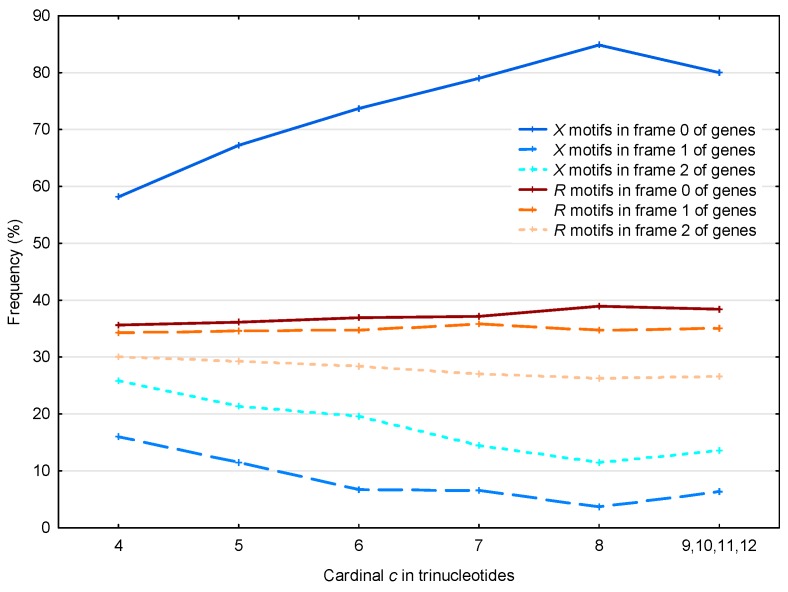
Proportion $P\left(X,c,f;{\mathbb{C}}_{g}\right)$ (%, Section 2.4) of the X motifs $m\left(X,c,f;{\mathbb{C}}_{g}\right)$ in the frames f=0 (reading frame; dark blue full line), f=1 (blue dashed line) and f=2 (light blue dotted line) of genes ${\mathbb{C}}_{g}$ in *S. cerevisiae*. Mean proportion $\bar{P}\left(R,c,f;{\mathbb{C}}_{g}\right)$ (%, Section 2.4) of the R motifs $m\left(R,c,f;{\mathbb{C}}_{g}\right)$ in the frames f=0 (reading frame; dark red full line), f=1 (red dashed line) and f=2 (light red dotted line) of genes ${\mathbb{C}}_{g}$ in *S. cerevisiae*. The abscissa shows the cardinality c=4,…,12 in trinucleotides. The ordinate gives the proportions P in percentage.

The 56,895 X motifs and the 39,247 R motifs in the *S. cerevisiae* genes ${\mathbb{C}}_{g}$ are analyzed according to their three frames (Figure 7).

First, if we consider the case of the R motifs, as expected their frequency is close to the random case of 1/3 in each frame of genes (one chance out of 3 to retrieve the reading frame). The observed frequency of R motifs in frame 2 is less than 1/3, which is related to the two facts that (i) there are more stop trinucleotides in frame 2 compared to frame 1 (Table 2); and (ii) the R motifs do not contain stop trinucleotides by construction (see Section 2.2). Indeed, among the 430,286 stop trinucleotides in the *S. cerevisiae* genes, 185,800 are located in frame 1 and 244,486 are located in frame 2.

In contrast, the X motifs present a non-random distribution, with 63% located in frame 0 (reading frame) of the genes (63% being also the average frequency of X motifs for all cardinalities in frame 0 in Figure 7). Again, we found the same correlation as that described in Section 3.1.2 (see Figure 5), namely that the effect is more pronounced for X motifs with large cardinalities. However, it is important to remember that the X motifs of low cardinalities are much more abundant.

Again in contrast to the R motifs, the X motifs occur preferentially in frame 2 compared to frame 1 with a significant difference of about 10%. Indeed, the observed average probability difference between the X motifs in frame 2 and the X motifs in frame 1 is equal to

$$\begin{array}{l} \bar{P\left(X,2;{\mathbb{C}}_{g}\right)-P\left(X,1;{\mathbb{C}}_{g}\right)}\\ \;=\frac{\sum_{c\geq 4}\left[\left(P\left(X,c,2;{\mathbb{C}}_{g}\right)-P\left(X,c,1;{\mathbb{C}}_{g}\right)\right)\left(N\left(X,c,1;{\mathbb{C}}_{g}\right)+N\left(X,c,2;{\mathbb{C}}_{g}\right)\right)\right]}{\sum_{c\geq 4}\left(N\left(X,c,1;{\mathbb{C}}_{g}\right)+N\left(X,c,2;{\mathbb{C}}_{g}\right)\right)}\\ \;=10.0\% \end{array}$$

where $P\left(X,c,f;{\mathbb{C}}_{g}\right)$ and $N\left(X,c,f;{\mathbb{C}}_{g}\right)$ with the frame f=1,2 are defined in Section 2.4.

This result is in agreement with the circular code theory. Indeed, a simple probabilistic model based on the independent occurrence of trinucleotides in reading frame 0 can estimate the real probabilities of the three circular codes X, $X_{1}$ and $X_{2}$ (Definition 8) observed in the shifted frames 1 and 2. Indeed, the estimated probabilities of X in frames 2 and 1 of eukaryotic genes equal to 29.4% and 25.5%, respectively, are identical (at the level of the percentage) to their corresponding probabilities in real sequences which are equal to 29.4% and 25.6%, respectively (Table 5b in [2]). This frequency asymmetry of the circular code X in frames 1 and 2 has been related to the frequency asymmetry of the circular codes $X_{1}$ and $X_{2}$ in frame 0. Indeed, in frame 0 of eukaryotic genes, the frequencies of the circular codes $X_{1}$ and $X_{2}$ are equal to 39.0% and 28.9%, respectively (Table 5b in [2]).

Since the frame 0 has no stop trinucleotides, the theoretical occurrence probability of the circular code X, with 20 trinucleotides, is equal to 20/64=31.25%. Similarly, the occurrence probability of the circular code $X_{1}$ (20 trinucleotides with one stop trinucleotide, TAG) is equal to 19/64=29.69%, and the occurrence probability of the circular code $X_{2}$ (20 trinucleotides with two stop trinucleotides, TAA and TGA) is equal to 18/64=28.13%. Thus, the probability difference between the two circular codes $X_{1}$ and $X_{2}$ is equal to 1/64=1.56%. We conclude that the frequency asymmetry of $X_{1}$ and $X_{2}$ in frame 0 cannot be explained solely by the presence of stop trinucleotides.

Although this frequency asymmetry of $X_{1}$ and $X_{2}$ has been identified in eukaryotic genes ([14], Figure 2 and Section 2.2; [15], Section 1.2.2) and prokaryotic genes ([16], Section 3.1.2), it has no biological explanation so far. However, it can explain the frequency asymmetry of the code X in frames 1 and 2. Thus, there is a strong correlation between the theoretical results of the three circular codes X, $X_{1}$ and $X_{2}$ in genes, i.e., three sets of 20 trinucleotides, described in the previous work and the results observed here with the circular code motifs. In the same way that the frequency asymmetry of $X_{1}$ and $X_{2}$ in frame 0 of genes is not explained from a biological point of view, the frequency asymmetry of X in frames 1 and 2 of genes is also not explained.

**Figure 8:**
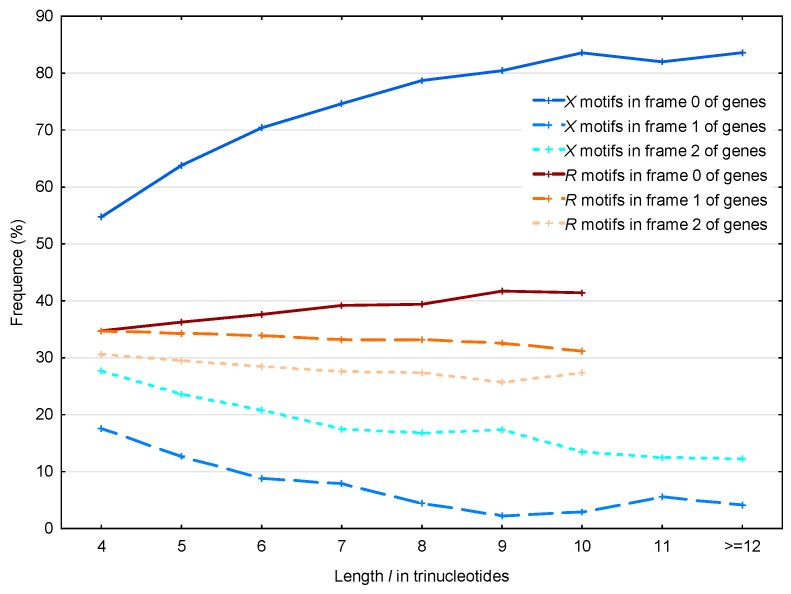
Proportion $P\left(X,l,f;{\mathbb{C}}_{g}\right)$ (%, Section 2.4) of the X motifs $m\left(X,l,f;{\mathbb{C}}_{g}\right)$ in the frames f=0 (reading frame; dark blue full line), f=1 (blue dashed line) and f=2 (light blue dotted line) of genes ${\mathbb{C}}_{g}$ in *S. cerevisiae*. Mean proportion $\bar{P}\left(R,l,f;{\mathbb{C}}_{g}\right)$ (%, Section 2.4) of the R motifs $m\left(R,l,f;{\mathbb{C}}_{g}\right)$ in the frames f=0 (reading frame; dark red full line), f=1 (red dashed line) and f=2 (light red dotted line) of genes ${\mathbb{C}}_{g}$ in *S. cerevisiae*. The abscissa shows the length l≥4 in trinucleotides. The ordinate gives the proportions P in percentage.

The same results are observed by analyzing the distribution of the 56,895 X motifs and the 39,247 R motifs in the *S. cerevisiae* genes as a function of their lengths (Figure 8). Note that we did not observe R motifs of length strictly greater than 10 trinucleotides.

The observed average probability difference with the X motifs in frames 2 and 1 is retrieved as a function of their length

$$\begin{array}{l} \bar{P\left(X,2;{\mathbb{C}}_{g}\right)-P\left(X,1;{\mathbb{C}}_{g}\right)}\\ \;=\frac{\sum_{l\geq 4}\left[\left(P\left(X,l,2;{\mathbb{C}}_{g}\right)-P\left(X,l,1;{\mathbb{C}}_{g}\right)\right)\left(N\left(X,l,1;{\mathbb{C}}_{g}\right)+N\left(X,l,2;{\mathbb{C}}_{g}\right)\right)\right]}{\sum_{l\geq 4}\left(N\left(X,l,1;{\mathbb{C}}_{g}\right)+N\left(X,l,2;{\mathbb{C}}_{g}\right)\right)}\\ \;=10.5\% \end{array}$$

where $P\left(X,l,f;{\mathbb{C}}_{g}\right)$ and $N\left(X,l,f;{\mathbb{C}}_{g}\right)$ with the frame f=1,2 are defined in Section 2.4.

### 3.3. Identification of S. cerevisiaeX Genes

In the following, we define an X gene to be a gene containing at least one X motif of cardinality c≥4 trinucleotides in any frame. A non-X gene is a gene with no X motif of cardinality c≥4 trinucleotides in any frame. In the genome of *S. cerevisiae*, 6175 genes out of 6691 contain X motifs (92.3%), while 516 genes do not contain X motifs (7.7%). The number of X motifs per gene varies from a single X motif, up to the gene “huge dynein-related AAA-type ATPase (midasin)” of length 14,732 nucleotides containing a series of 67 X motifs.

**Figure 9:**
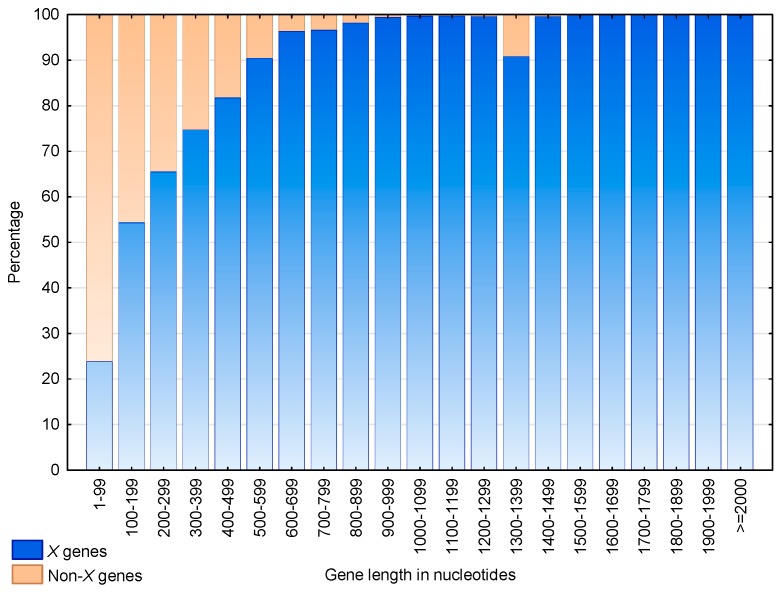
Proportion of X genes (blue) and non-X genes (braun) according to their nucleotide length in *S. cerevisiae*. An X gene is a gene containing at least one X motif of cardinality c≥4 trinucleotides in any frame. A non-X gene is a gene with no X motif of cardinality c≥4 trinucleotides in any frame. The abscissa shows the gene length in intervals of 100 nucleotides. The ordinate gives the percentage of genes.

Figure 9 shows the distributions of the X genes and non-X genes according to their lengths. The proportion of X genes increases with their length. Indeed, more than 50% of the genes of length >200 nucleotides and more than 90% of the genes of length >500 nucleotides are X genes. Nevertheless, an anomaly is observed for genes of length 1300–1399, where 27 out of the 266 genes (i.e., 10.2%) are not X genes. A functional analysis showed that these 27 non-X genes are in fact retrotransposons of viral origin.

This observation led us to perform a more detailed study of the functional annotations associated with the *S. cerevisiae* genes, as shown in Table 3. In the SGD database, 5383 genes have a status of “Verified” genes, meaning that experimental evidence exists and that a gene product is produced in *S. cerevisiae*; 546 genes have a status of “Uncharacterized” genes, implying that they are likely to encode expressed proteins, as suggested by the existence of orthologs in one or more other species, but for which there are no specific experimental data demonstrating that a gene product is produced in *S. cerevisiae*; 673 genes have a “Dubious” status meaning that they are unlikely to encode an expressed protein. Dubious genes may meet some or all of the following criteria: (i) the gene is not conserved in other *Saccharomyces* species; (ii) there is no well-controlled, small-scale, published experimental evidence that a gene product is produced; (iii) a phenotype caused by disruption of the gene can be ascribed to mutation of an overlapping gene; and (iv) the gene does not contain an intron. Finally, 89 genes are transposons, including any of the five classes (TY1 through TY5) of mobile genetic elements in yeast that contain long terminal repeats flanking a central epsilon element that encodes two gene products.

The proportion of X genes and non-X genes strongly depends on their status. For example, 97.8% of verified genes are X genes, 82.2% of uncharacterized genes are X genes while only 60.0% of dubious genes are X genes, in agreement with the experimental evidence available.

Thus, the presence–absence of X motifs in a gene is an important and new factor in the classification of genes as functional or not as shown by the following conditional probabilities deduced from Table 3:
P(Non-verified genes | Non-X genes)=(97+269+29)/516=395/516=76.6%P(Verified genes | Non-X genes)=121/516=23.4%P(Verified genes | X genes with≥1 X motifs)=5262/6175=85.2%P(Verified genes | X genes with ≥2 X motifs)=5082/5737=88.6%P(Verified genes | X genes with≥3 X motifs)=4758/5217=91.2%P(Verified genes | X genes with ≥4 X motifs )=4388/4729=92.8%P(Verified genes | X genes with ≥5 X motifs)=4013/4278=93.8%
the non-verified genes being the uncharacterized and dubious genes, and the transposable elements.

Clearly, the probability of verified genes in the set of genes with ≥n X motifs increases as n increases. However, the biggest difference in conditional probabilities of verified genes is observed for genes with no X motifs compared to genes with ≥1X motifs, and therefore we retain our definition of an X gene as a gene containing at least one X motif in the remainder of this article.

### 3.4. Trinucleotide Composition in the X Motifs of S. cerevisiae Genes

We compared the trinucleotide composition of the 5262 *S. cerevisiae* verified X genes with the composition of the X motifs in frame 0 of these genes (Table 4) and found that they are highly similar (correlation coefficient r=0.99).

As the length of the 5262 *S. cerevisiae* verified X genes is 2,719,966 trinucleotides, the coverage of X genes by the X motifs is equal to 154,635/2,719,966=5.7%.

## 4. Conclusions

The theory of the circular code X in genes has been developed using a combinatorial approach since 1996. For the first time, we tested this theory by analysing the X motifs, i.e., motifs from this circular code X, in the complete genome of the yeast *S. cerevisiae*. This organism was chosen because it has been a “model” organism for many years, the genome is relatively small and compact, and the genes generally have a simple intron/exon structure.

The main result demonstrated is a significant enrichment of X motifs in the reading frame of genes of *S. cerevisiae* (see results in Section 3.1–Section 3.2). Furthermore, the statistical distribution of X motifs in the three frames of *S. cerevisiae* genes, in particular the preferential occurrence of X motifs in frame 2 compared to frame 1 (see results in Section 3.2), is in agreement with the circular code theory concerning the well-known frequency asymmetry of the circular codes $X_{1}$ and $X_{2}$ in prokaryotic and eukaryotic genes ([14], Figure 2 and Section 2.2; [15], Section 1.2.2; [16], Section 3.1.2).

The longest X motifs in the genes of *S. cerevisiae* are of length greater than 100 nucleotides. Surprisingly, these X motifs exhibit two structures fundamentally different (Table 1). The 1st class is exemplified by X motifs containing a sequence of a repeated trinucleotide ${\left(N_{1}N_{2}N_{3}\right)}^{n}$, while the 2nd class is represented by X motifs with no repeated trinucleotides (n=1). An intermediary class is composed of X motifs between these two extremes, i.e., composed of a series of different short trinucleotide repeats. Half of the *S. cerevisiae* genes with very long X motifs have paralogues that arose from the whole genome duplication (WGD) event that occurred in an ancestor of *S. cerevisiae* ~100 million years ago [17], even though ~80% of the duplicated genes have since been lost [17]. Furthermore, the functional annotations found in the SGD database indicate that many of the genes with very long X motifs encode important physiological polypeptides involved in, for example, transport from the Golgi, chromatin modelling or are located in the mitochondria.

We have shown that the presence of X motifs in a potential open reading frame can be used to predict whether the gene is likely to encode a functional protein. Indeed, X motifs are found in 98% of verified genes, while only 60% of dubious genes contain X motifs (see results in Section 3.3). Additional parameters related to the genes themselves or the structure, the length and positions of X motifs may improve the prediction accuracy in the future.

The question remains of whether the X motifs are simply the evolutionary relics of a primordial code that might have existed in the early stages of cellular life, or do they represent functional elements of the complex genome decoding system in extant organisms?

There seems to be a consensus that the standard genetic code conserves vestiges of earlier, simpler codes, that may have been used to code fewer amino acids than the modern set of 20. Many examples of such ancient genetic codes have been proposed, including the codes RRY of size 8 [18] and RNY of size 16 [19,20] (R={A,G}, Y={C,T}, N={A,C,G,T}), the codes GNC of size 4 and SNS of size 16 [21], and GHN of size 12 [22] (S={C,G}, H={A,C,T}), etc. All these codes are circular, with the exception of the SNS code (as, for example, CCC∈SNS). The codes RRY, RNY, GNC and GHN also belong to the more restrictive class of comma-free codes (longest path length l=2 in their associated graphs G(RRY), G(RNY), G(GNC) and G(GHN), details in [23]). The code RRY is in addition strong comma-free (longest path length l=1 in its associated graph G(RRY), details in [23]). The comma-free codes RRY and GHN are not self-complementary (as C(RRY)=RYY and C(GHN)=NDC with D={A,G,T}), while the codes RNY and GNC are self-complementary (as C(RNY)=RNY and C(GNC)=GNC). The comma-free code RNY can be decomposed into two subcodes of size 8 each which are both strong comma-free and complementary to each other (Proposition 3.28 in [23]) and almost included in the circular code X (Table 3a in [3]). Today, the genetic code has become too complex to use strong comma-free codes and comma-free codes (in the sense of having strong error-detecting properties, i.e., recognizing a frameshift immediately), and therefore, we suggest that nature moved on to the weaker circular codes.

Numerous hypotheses have been formulated concerning the evolution of the ancient genetic codes into the modern standard genetic code (reviewed in [24]). For example, several lines of evidence have been used to classify the standard 20 amino acids into 'early' and 'late' ones. Ten early amino acids (*EAA*) have been consistently identified in prebiotic chemistry experiments as well as in meteorites, in the following order of abundance: <Gly,Ala,Asp,Glu,Val,Ser,Ile,Leu,Pro,Thr> (reviewed in [24]). The ten late amino acids are entirely biogenic and were probably recruited into the code after the evolution of the respective biosynthetic pathways, possibly in complementary pairs. The circular code X encodes 12 amino acids, of which 8 correspond to these early amino acids, with the exception of *Ser* and *Pro*. Furthermore, a (ordered) subcode X′ of 10 trinucleotides among the 20 trinucleotides of X

$$X^{\prime }=<\{GGC,GGT\},GCC,\left\{GAC,GAT\right\},GAG,GTC,ATC,CTC,ACC>$$

codes 8 (ordered) early amino acids of the ten

EAA=<Gly,Ala,Asp,Glu,Val,Ile,Leu,Thr>.

The circular code $X^{\prime }$ is $C^{3}$ self-complementary. This ancient code $X^{\prime }$ is not comma-free as the longest path length l=4>2 in its associated graph $G\left(X^{\prime }\right)$. This result may suggest that the ancestral circular codes of X are also $C^{3}$ self-complementary. 

**Figure 10:**
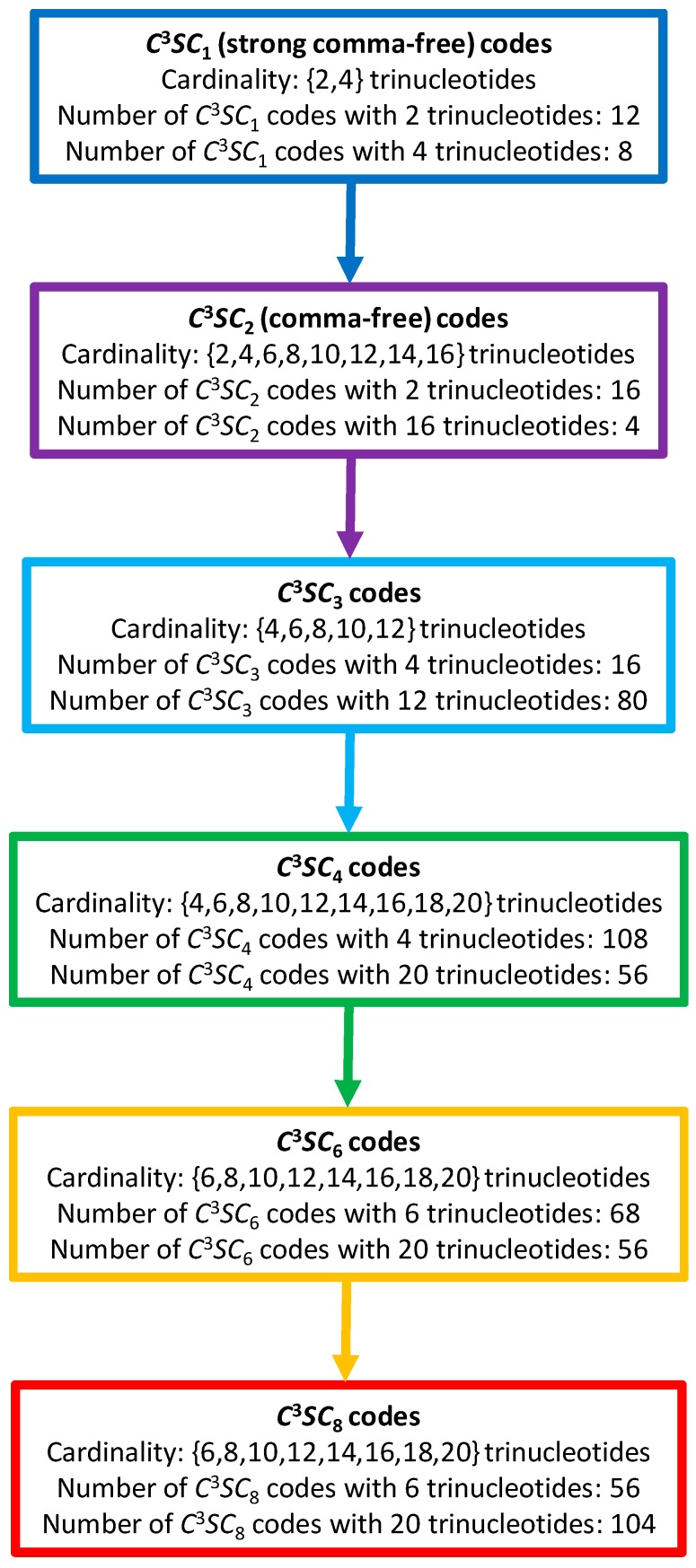
A model of the evolution of $C^{3}$ self-complementary circular codes. A $C^{3}SC_{l}$ code stands for a $C^{3}$ Self-complementary Circular code of longest path length l∈{1,2,3,4,6,8}. The maximal $C^{3}$ self-complementary trinucleotide circular code X (1) belongs to the class $C^{3}SC_{8}$ of cardinality 20 trinucleotides (red rectangle). A (ordered) non-maximal $C^{3}$ self-complementary trinucleotide circular code $X^{\prime }=<\{GGC,GGT\},GCC,\left\{GAC,GAT\right\},GAG,GTC,ATC,CTC,ACC>$ of 10 trinucleotides among the 20 trinucleotides of X belonging to the class $C^{3}SC_{4}$ of cardinality 10 trinucleotides (green rectangle) codes the 8 (ordered) early amino acids EAA=<Gly,Ala,Asp,Glu,Val,Ile,Leu,Thr>.

A model of the evolution of $C^{3}$ self-complementary circular codes can be proposed (Figure 10). We will use the following abbreviation in the following to classify these circular codes: a $C^{3}SC_{l}$ code stands for a $C^{3}$ Self-complementary Circular code of longest path length l∈{1,2,3,4,6,8}, l=5,7 being excluded (see Theorem 4.2 given for self-complementary circular codes in [25]). According to this model, the evolution of $C^{3}SC_{l}$ codes is based on an increase in combinatorial flexibility (number of codes, cardinality of codes, nucleotide window length of reading frame retrieval), starting with the strong comma-free codes ($C^{3}SC_{1}$ codes) with the strongest error-detecting properties, then the comma-free codes ($C^{3}SC_{2}$ codes) with strong error-detecting properties, then the $C^{3}SC_{3}$, $C^{3}SC_{4}$ and $C^{3}SC_{6}$ codes with low error-detecting properties, up to the $C^{3}SC_{8}$ codes with the lowest error-detecting properties, such as the circular code X found in extant genes. Note that the 216 $C^{3}$ self-complementary circular codes are the sum of the 56 $C^{3}SC_{4}$ codes plus the 56 $C^{3}SC_{6}$ codes plus the 104 $C^{3}SC_{8}$ codes. This combinatorial circular code evolution may also be associated with time evolution where strong comma-free codes and comma-free codes are more ancestral than circular codes. So, the circular code $X^{\prime }$ ($C^{3}SC_{4}$ of cardinality 10 trinucleotides) may be an intermediate between the ancient strong comma-free and comma-free codes ($C^{3}SC_{1}$ and $C^{3}SC_{2}$ codes), and the circular code X ($C^{3}SC_{8}$ code of cardinality 20 trinucleotides) in extant organisms.

The X motifs observed in the genes of *S. cerevisiae* may have retained a functional role in translation. Indeed, it has been observed previously that short X motifs have also been conserved in many transfer RNAs (tRNAs) and ribosomal RNAs (rRNAs) [26,27,28,29]. In particular, the universally conserved nucleotides A1492, A1493 and G530 in the ribosome decoding center are located in short X motifs. Understanding the pairing between the X motifs in genes and the short X motifs of the ribosome decoding center could shed light on the biological function of the circular code X in the genome decoding system of extant organisms. Furthermore, if X motifs do play a functional role, then mutations in these regions that lead to the loss of the X motif properties could have deleterious effects and may even be the cause of genetic diseases. In particular, long X motifs with repeats of certain trinucleotides could generate secondary structures that may be problematic in translation [30]. The effect of mutations in X motifs will be investigated in future work.

## Figures and Tables

**Table 1:** Longest X motifs in the genes ${\mathbb{C}}_{g}$ of *S. cerevisiae*. The 1st column gives the chromosome number, the 2nd, 3rd, 4th and 5th indicate the name, the start position, the end position and the nucleotide length, respectively, of genes containing the longest X motifs, the 6th, 7th and 8th point out the start position, the end position and the nucleotide length, respectively, of the longest X motifs, and 9th column gives the sequence of the longest X motifs.

Chr	Gene Name	Gene Start	Gene End	Gene Length	X Motif Start	X Motif End	X Motif Length	X Motif
VIII	YHR131C	365,340	367,892	2553	365,358	365,489	132	$m_{1}={\left(ATC\right)}^{3},GTC,ATC,{\left(GTC\right)}^{3},{\left(ATC\right)}^{7},{\left(GTC\right)}^{3},TTC,{\left(GTC\right)}^{3},{\left(ATC\right)}^{5},{\left(GTC\right)}^{4},{\left(ATC\right)}^{3},CTC,ACC,{\left(ATC\right)}^{2},ACC,GTC,ACC,{\left(GTC\right)}^{2},CTC$
XVI	YPL190C	185,317	187,725	2409	187,303	187,428	126	$m_{2}=GTT,GTC,GTT,GCC,{\left(TTC\right)}^{10},{\left(ATC\right)}^{2},ATC,{\left(GTC\right)}^{2},{\left(ATC\right)}^{4},GTC,{\left(ATC\right)}^{2},CTC,{\left(TTC\right)}^{2},CTC,{\left(TTC\right)}^{2},CTC,TTC,ATT,{\left(ATC\right)}^{3},GTC,$${\left(ATC\right)}^{2},ATT$
XVI	YPL158C	252,034	254,310	2277	252,241	252,363	123	$m_{3}={\left(TTC\right)}^{5},{\left(ATC\right)}^{2},TTC,{\left(ATC\right)}^{2},{\left(TTC\right)}^{2},ATC,{\left(TTC\right)}^{2},ATC,{\left(TTC\right)}^{2},{\left(ATC\right)}^{2},ATT,{\left(TTC\right)}^{6},{\left(ATC\right)}^{2},TTC,{\left(ATC\right)}^{2},TTC,ATC,{\left(TTC\right)}^{4},$CTC,GTC,GGC
XVI	YPR042C	650,435	653,662	3228	650,504	650,611	108	$m_{4}={\left(ATT\right)}^{14},GTT,ATT,GTT,{\left(ATT\right)}^{3},GTT,ATC,ATT,ATC,{\left(ATT\right)}^{2},GTT,GTA,GTT,ATT,GGT,{\left(ATT\right)}^{3},GTT,ATT$
VII	YGL150C	221,104	225,573	4470	224,830	224,934	105	$m_{5}=GTC,ATT,GTC,ATT,TTC,ATT,TTC,GTT,GTC,GTT,GTC,GTT,GTC,{\left(GTT\right)}^{3},TTC,ATT,GTC,GTT,GTC,GTT,GTC,TTC,GTC,TTC,$ACC,GTT,ATT,GCC,ATC,CTC,GTT,TTC,GTC
II	YBR150C	541,209	544,493	3285	541,446	541,550	105	$m_{6}={\left(ATC\right)}^{3},ATT,GTC,{\left(ATC\right)}^{20},ATT,AAT,ATT,GTT,GTC,ATT,GTC,{\left(ATT\right)}^{2},GTT$
XI	YKR072C	576,435	578,123	1689	576,471	576,572	102	$m_{7}=TTC,GTC,CTC,GTC,CTC,GTC,{\left(ATC\right)}^{2},{\left(GTC\right)}^{13},ATC,{\left(GTC\right)}^{2},ATC,GTC,{\left(ATC\right)}^{3},{\left(GTT\right)}^{3},TTC,GTT$
XII	YLR114C	374,944	377,238	2295	375,259	375,360	102	$m_{8}=ATC,GCC,ATT,TTC,ATC,GCC,CTC,ACC,GTC,ATC,GCC,ATT,TTC,ATC,GCC,CTC,ACC,GTC,ATC,GCC,ATT,TTC,ATC,GCC,CTC,$ACC,GTC,ATC,GTC,ATC,GTC,ATC,GTC,CTC

**Table 2:** Number of stop trinucleotides {TAA,TAG,TGA} in frames 1 and 2 of the genes ${\mathbb{C}}_{g}$ in *S. cerevisiae*.

	Frame 1	Frame 2	Total
TAA	64,458	91,661	156,119
TAG	51,774	37,366	89,140
TGA	69,568	115,459	185,027
Total	185,800	244,486	430,286

**Table 3:** Numbers of X genes and non-X genes depending on the status of *S. cerevisiae* genes according to the SGD database. An X gene is a gene containing at least one X motif of cardinality c≥4 trinucleotides in any frame. A non-X gene is a gene with no X motifs of cardinality c≥4 trinucleotides in any frame. The total column represents the sum of X genes with ≥1 X motifs and the non-X genes, i.e., the number of *S. cerevisiae* genes in each category.

	X Genes with X Motifs					Non-X Genes	Total
	≥1	≥2	≥3	≥4	≥5		
Verified genes	5262	5082	4758	4388	4013	121	5383
Uncharacterized genes	449	348	266	221	174	97	546
Dubious genes	404	247	133	61	32	269	673
Transposable elements	60	60	60	59	59	29	89
Total	6175	5737	5217	4729	4278	516	6691

**Table 4:** Trinucleotide compositions in the 5262 *S. cerevisiae* verified X genes and in the X motifs in frame 0 of these genes.

	X Motifs		Verified X Genes	
	Number	%	Number	%
AAC	9796	6.33	48,354	6.27
AAT	13,228	8.55	71,108	9.22
ACC	5245	3.39	24,307	3.15
ATC	7569	4.89	33,049	4.29
ATT	12,117	7.84	58,617	7.60
CAG	4350	2.81	24,378	3.16
CTC	2499	1.62	10,475	1.36
CTG	4121	2.66	20,695	2.68
GAA	15,353	9.93	90,008	11.68
GAC	9125	5.90	39,699	5.15
GAG	7935	5.13	38,265	4.96
GAT	14,132	9.14	74,274	9.64
GCC	4896	3.17	23,549	3.05
GGC	3992	2.58	18,951	2.46
GGT	9004	5.82	44,365	5.76
GTA	4623	2.99	23,497	3.05
GTC	5132	3.32	21,884	2.84
GTT	8538	5.52	42,051	5.46
TAC	5983	3.87	28,452	3.69
TTC	6997	4.52	34,862	4.52
Total	154,635	100.00	770,840	100.00

## Appendix A. Random Codes

30 random codes R are generated according to the properties of the maximal $C^{3}$ self-complementary trinucleotide circular code X (1), except its circularity property:

(i) R has a cardinality equal to 20 trinucleotides;
(ii) The total number of each nucleotide A, C, G and T in R is equal to 15;
(iii) R has no stop trinucleotides {TAA,TAG,TGA} and no periodic trinucleotides {AAA,CCC,GGG,TTT};
(iv) R is not a circular code. Its associated graph G(R) is cyclic (G(R) being not shown).

$R_{1}=\left\{AAC,AAT,ACA,ACT,AGG,ATA,ATT,CAA,CAG,CTC,CTG,GCC,GCG,GCT,GGC,GTA,GTC,GTT,TGC,TGT\right\}$
$R_{2}=\left\{ACT,AGG,AGT,ATA,ATG,CAA,CAG,CCA,CCG,CTC,GAA,GAG,GCT,GGC,TAC,TAT,TCC,TCT,TGG,TTG\right\}$
$R_{3}=\left\{AAT,ACC,AGA,ATT,CCT,CGA,CGG,CTA,CTC,CTG,GAA,GAC,GAT,GCC,GCG,GGT,GTG,TAC,TAT,TTA\right\}$
$R_{4}=\left\{ACC,AGA,AGG,ATA,ATC,CCG,CCT,CGC,CTA,CTC,CTG,CTT,GAA,GAT,GCA,GGA,GTA,GTG,TGT,TTA\right\}$
$R_{5}=\left\{AAC,AAG,ACA,AGC,CCA,CGA,CTG,CTT,GAG,GCA,GCC,GGC,GTA,GTT,TAC,TAT,TCA,TCT,TGG,TGT\right\}$
$R_{6}=\left\{AAC,ACG,AGA,AGG,ATA,CCT,CGC,CGG,CGT,CTA,CTG,GAA,GAC,GCT,GTA,TAT,TCC,TGC,TTA,TTG\right\}$
$R_{7}=\left\{AAT,ACT,AGT,ATC,CAA,CCT,CGA,CTG,GAC,GAG,GAT,GCA,GCG,GCT,GTA,TAC,TAT,TCC,TCG,TGG\right\}$
$R_{8}=\left\{AAG,ACG,AGG,ATA,ATG,CAT,CCA,CGG,CGT,CTT,GAC,GCA,GCC,GCT,GGC,TAC,TAT,TCA,TTA,TTG\right\}$
$R_{9}=\left\{AAG,ACG,ACT,AGG,AGT,ATC,CAA,CAG,CCT,CGA,CGC,CTG,CTT,GCA,GGT,GTG,GTT,TAC,TAT,TCA\right\}$
$R_{10}=\left\{ACC,ACT,AGA,AGT,ATG,CAG,CAT,CCA,CGC,CTT,GAA,GAT,GCA,GCT,GGA,GGC,GTA,TCT,TGT,TTC\right\}$
$R_{11}=\left\{AAC,AAT,ACA,ACT,AGG,AGT,ATA,CAT,CGC,CTA,CTC,GAC,GCA,GCC,GGC,GTC,GTT,TGG,TGT,TTG\right\}$
$R_{12}=\left\{AAG,ACG,ACT,AGG,ATA,CAA,CAC,CAG,CCT,CGT,CTA,GAG,GCA,GCC,GTG,GTT,TAT,TCG,TCT,TTG\right\}$
$R_{13}=\left\{AAC,AAT,AGA,AGC,ATT,CAG,CCT,CGC,CGG,CTA,GAC,GAT,GCC,GGC,GGT,GTA,TAT,TCA,TTC,TTG\right\}$
$R_{14}=\left\{ACA,ACC,ACG,AGA,AGG,ATC,ATT,CAC,CAT,CCT,CGT,GAG,GCA,GGA,GGC,GTT,TGC,TGT,TTA,TTC\right\}$
$R_{15}=\left\{AAC,ACA,ACC,AGC,AGG,ATA,CAT,CGC,CGG,CTT,GCA,GGA,GTA,GTG,TAC,TCG,TCT,TGG,TTA,TTC\right\}$
$R_{16}=\left\{AAC,ACC,ACG,ACT,AGA,AGG,ATA,ATC,ATG,CAA,CCG,CCT,CGT,GCG,GTG,TCG,TGG,TTA,TTC,TTG\right\}$
$R_{17}=\left\{AAC,AAG,ACA,ACG,ACT,AGC,AGG,CAT,CGA,CTA,CTC,GAG,GGA,GTT,TCC,TCT,TGC,TGG,TTC,TTG\right\}$
$R_{18}=\left\{AAT,ACT,AGC,AGG,ATA,ATC,ATG,CAA,CGC,CTA,CTC,GAG,GCC,GCT,GTA,GTC,GTT,TAC,TCG,TGG\right\}$
$R_{19}=\left\{AAC,AGA,AGG,AGT,ATA,CAG,CCA,CGA,CGG,CTA,CTG,CTT,GAC,GGA,GTC,GTT,TAT,TCC,TCG,TTC\right\}$
$R_{20}=\left\{AAT,AGA,AGG,CAA,CAG,CCA,CGG,CTC,CTG,GAC,GCA,GCT,GTA,GTC,GTG,TAC,TAT,TCA,TTC,TTG\right\}$
$R_{21}=\left\{AAG,ACA,ACC,ACT,ATA,ATG,ATT,CAA,CAT,CGA,CGG,CTG,CTT,GAG,GCG,GCT,GGC,GTG,TCT,TTC\right\}$
$R_{22}=\left\{ACA,AGG,AGT,ATG,ATT,CAT,CCG,CGA,CTC,CTT,GAA,GAC,GAG,GCA,GCG,GTA,TAC,TCT,TGC,TTC\right\}$
$R_{23}=\left\{AAC,ACA,ACT,AGC,ATA,CAA,CAT,CCG,CTT,GAA,GAC,GCG,GCT,GGT,GTA,GTC,TCG,TCT,TGG,TGT\right\}$
$R_{24}=\left\{ACA,AGA,AGT,ATA,ATC,CAG,CAT,CCT,CGC,CTA,CTC,GAG,GCG,GGA,GTA,TAC,TCG,TCT,TGG,TGT\right\}$
$R_{25}=\left\{ACG,ACT,AGG,ATC,CAA,CAG,CAT,CGA,CTG,GAT,GCC,GCG,GGT,GTA,GTC,TAC,TAT,TCA,TGC,TTA\right\}$
$R_{26}=\left\{AAT,ATC,ATG,CAA,CAC,CAG,CAT,CGG,CGT,CTA,GAC,GAG,GCG,GGC,GTA,TAT,TCA,TCT,TGC,TGT\right\}$
$R_{27}=\left\{ACA,ACC,ACG,AGT,ATG,CAG,CTA,CTG,GAC,GCA,GCC,GCT,GGA,GTA,GTC,GTT,TAT,TCA,TCG,TTA\right\}$
$R_{28}=\left\{AAC,AAT,ACA,AGG,AGT,ATC,ATT,CAA,CAC,CAG,CCG,CGG,CTG,CTT,GAT,GCG,GGT,TCG,TCT,TGT\right\}$
$R_{29}=\left\{ACA,AGG,AGT,ATC,ATG,CCG,CGA,CTA,GAG,GCA,GCG,GGA,GTA,TAC,TCA,TCC,TCG,TCT,TTA,TTC\right\}$
$R_{30}=\left\{AAG,AAT,ACC,ATA,ATT,CAA,CAC,CCA,CGG,CGT,CTG,GAC,GCA,GCG,GGC,GTC,GTT,TAT,TGT,TTG\right\}$
